# Supplementary figures and images for: MSPA-informed SLEUTH urban growth modeling for green space protection in Ottawa
Source: PLoS One. 2025 Aug 8;20(8):e0328656. doi: 10.1371/journal.pone.0328656 (PMC12334021; doi:10.1371/journal.pone.0328656)

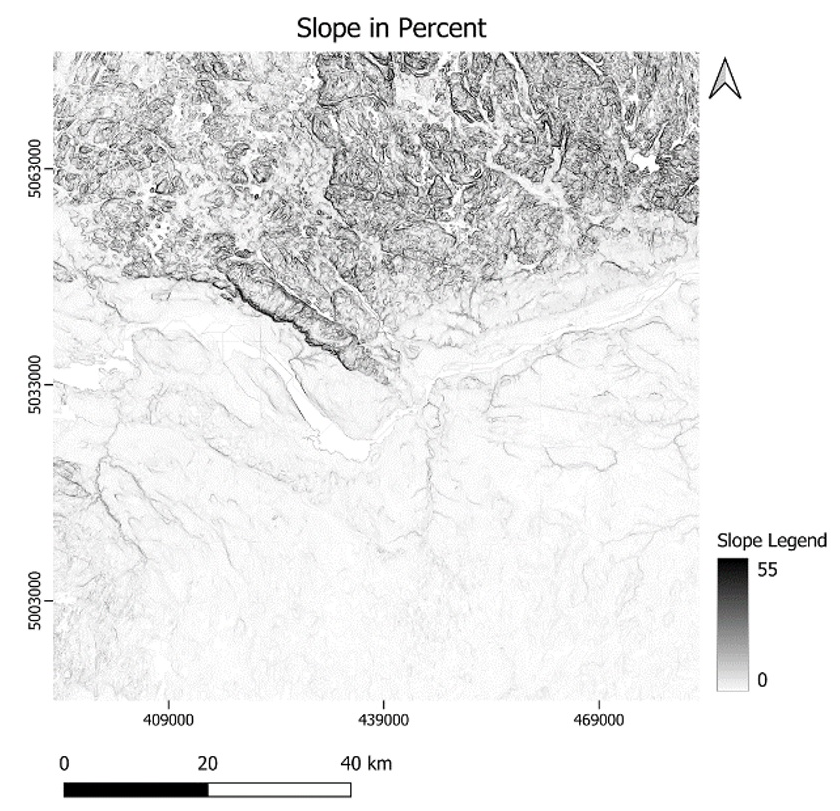

Supplement: S1 Fig — (TIF) [file pone.0328656.s001.tif]

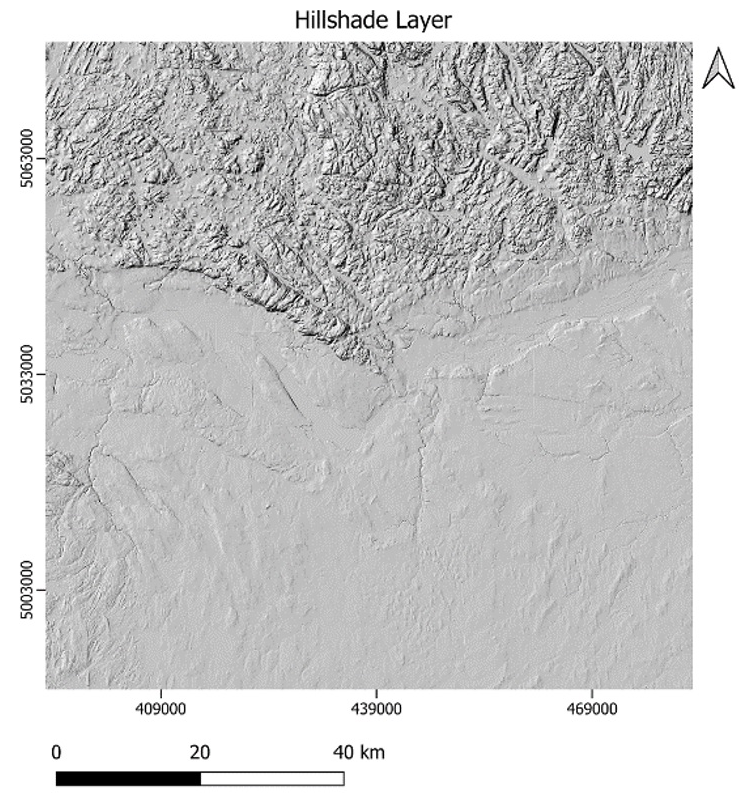

Supplement: S2 Fig — (TIF) [file pone.0328656.s002.tif]

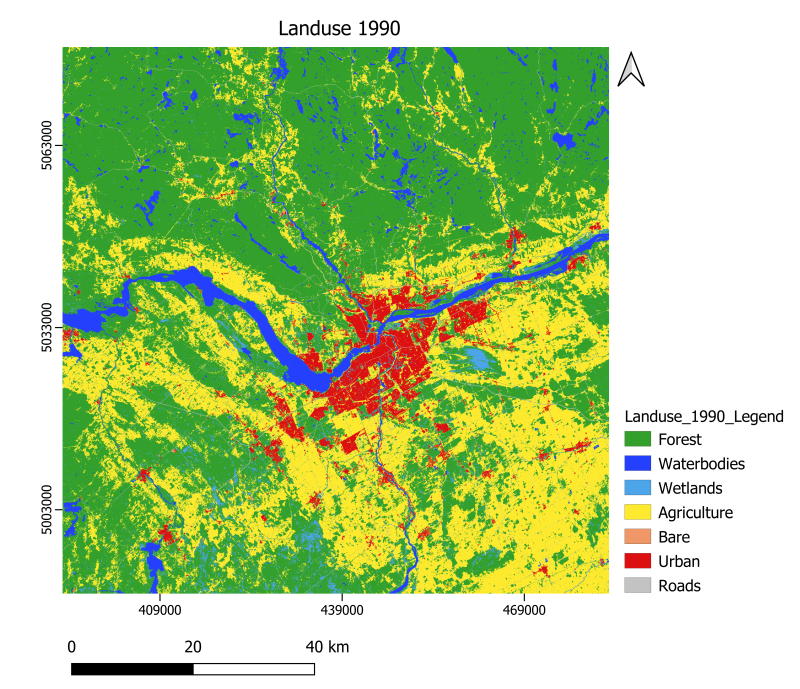

Supplement: S3 Fig — (TIF) [file pone.0328656.s003.tif]

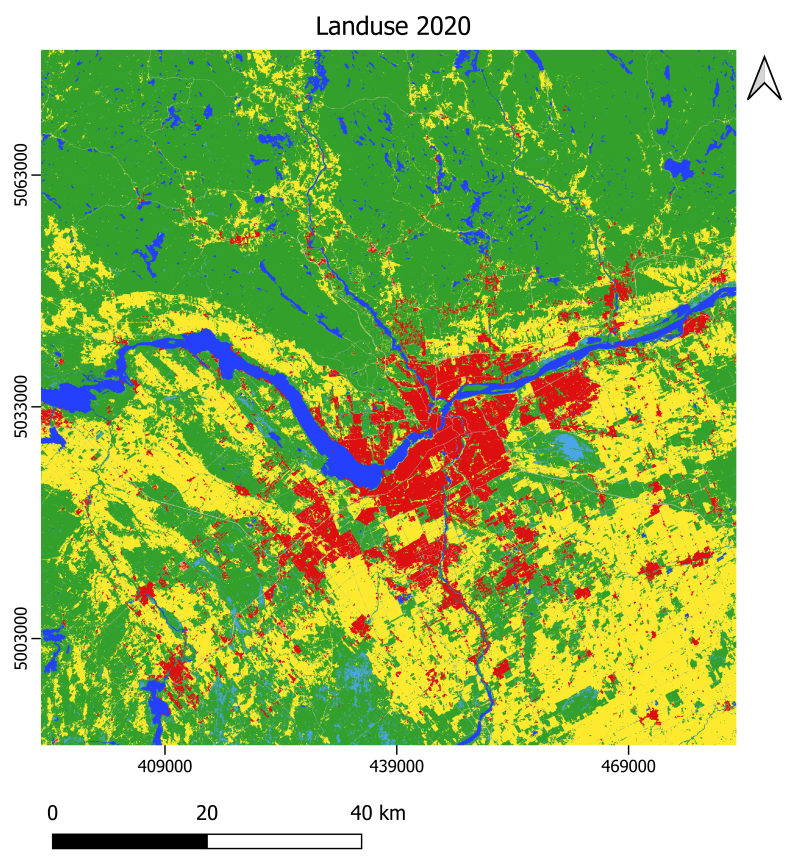

Supplement: S4 Fig — (TIF) [file pone.0328656.s004.tif]

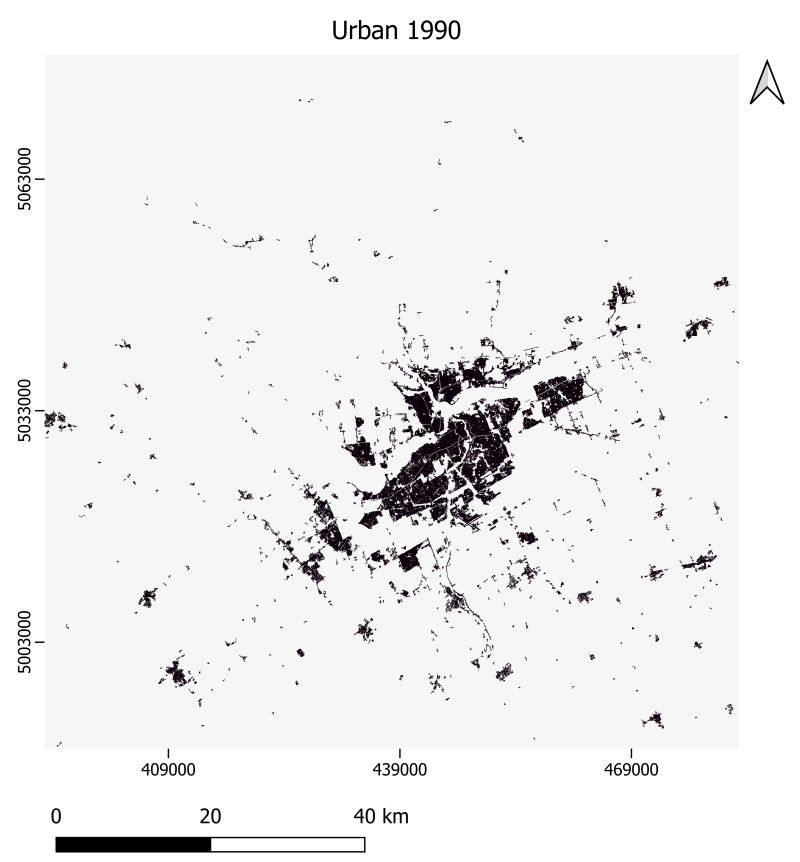

Supplement: S5 Fig — (TIF) [file pone.0328656.s005.tif]

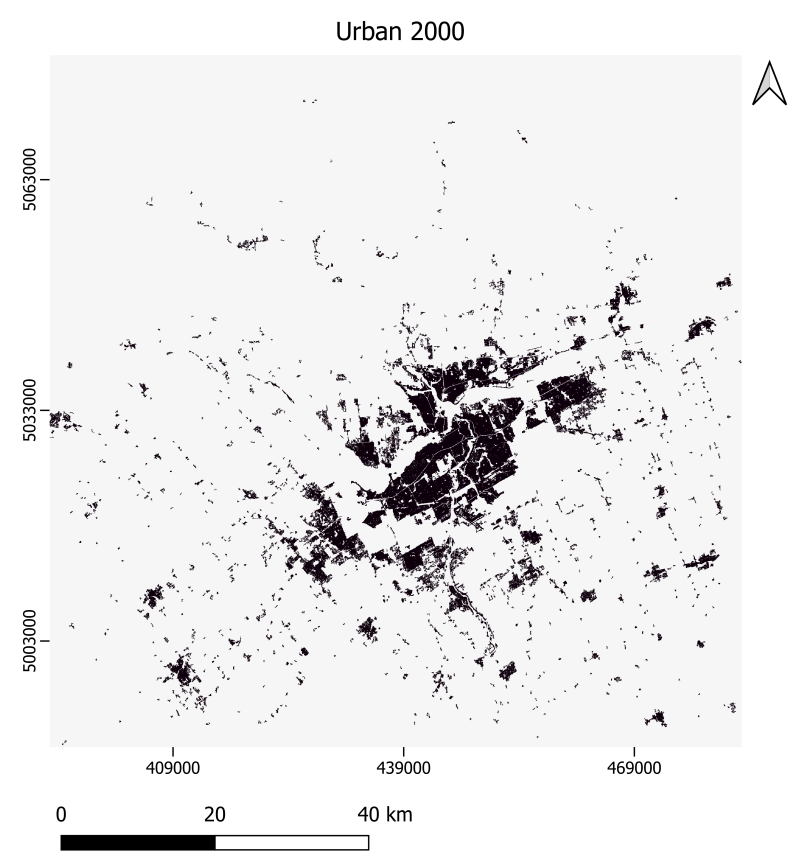

Supplement: S6 Fig — (TIF) [file pone.0328656.s006.tif]

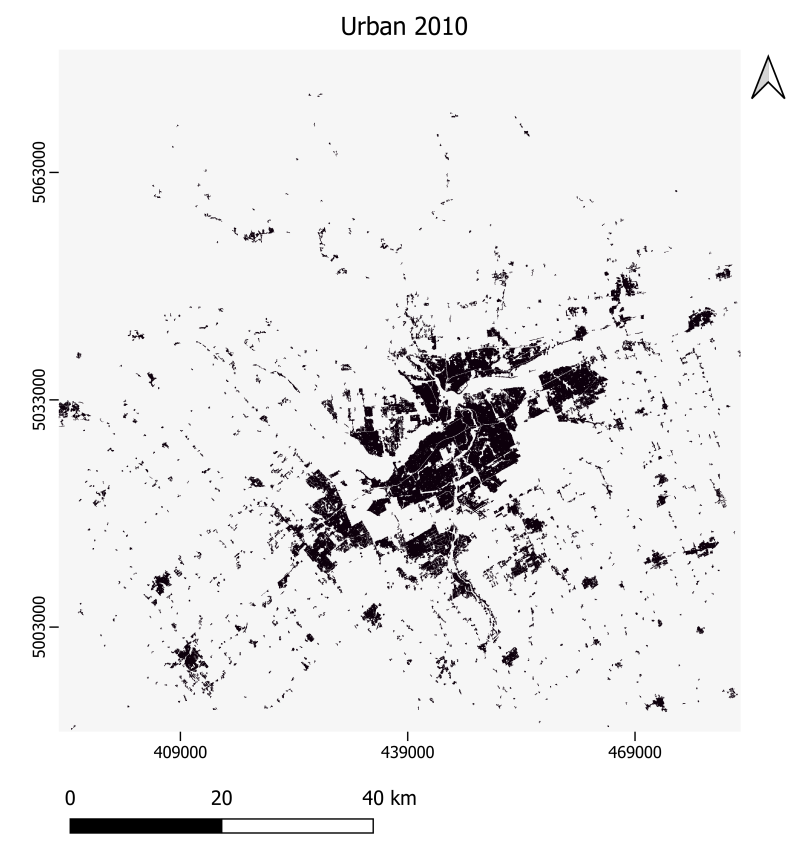

Supplement: S7 Fig — (TIF) [file pone.0328656.s007.tif]

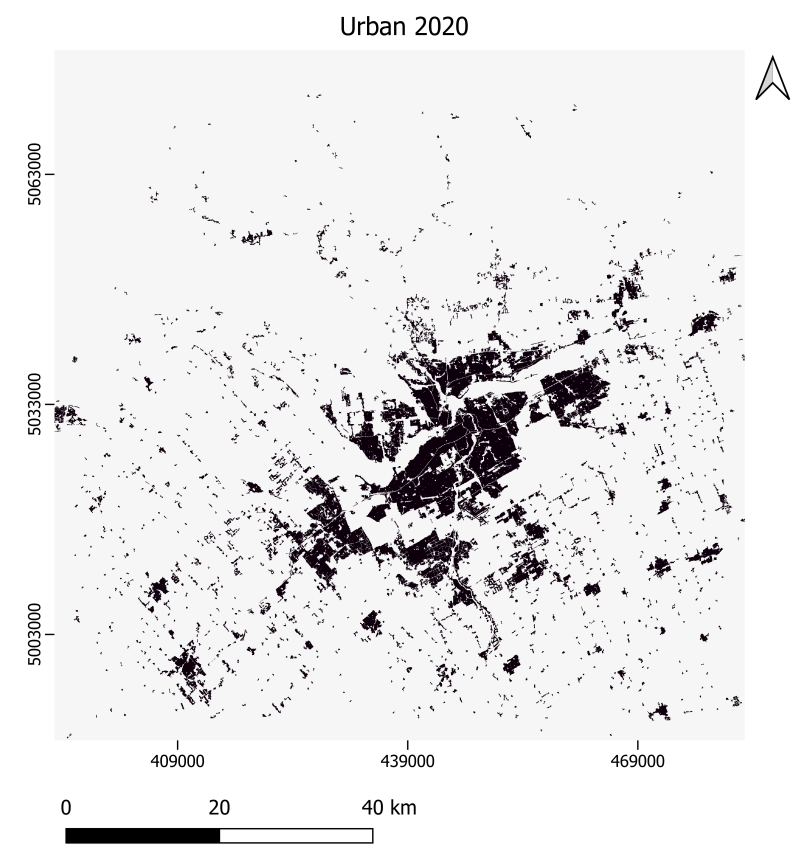

Supplement: S8 Fig — (TIF) [file pone.0328656.s008.tif]

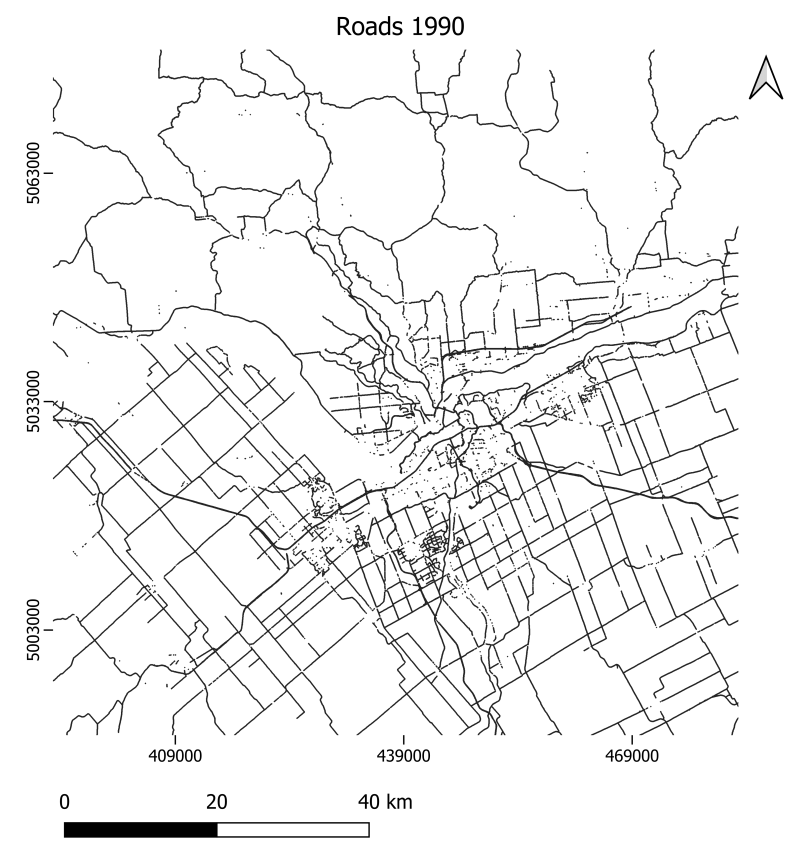

Supplement: S9 Fig — (TIF) [file pone.0328656.s009.tif]

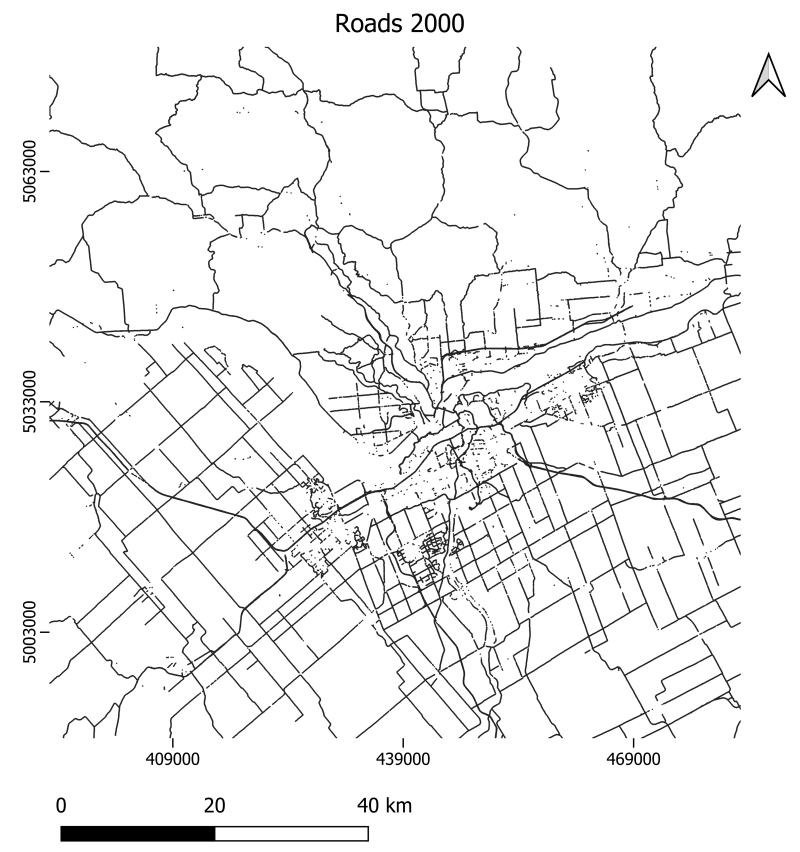

Supplement: S10 Fig — (TIF) [file pone.0328656.s010.tif]

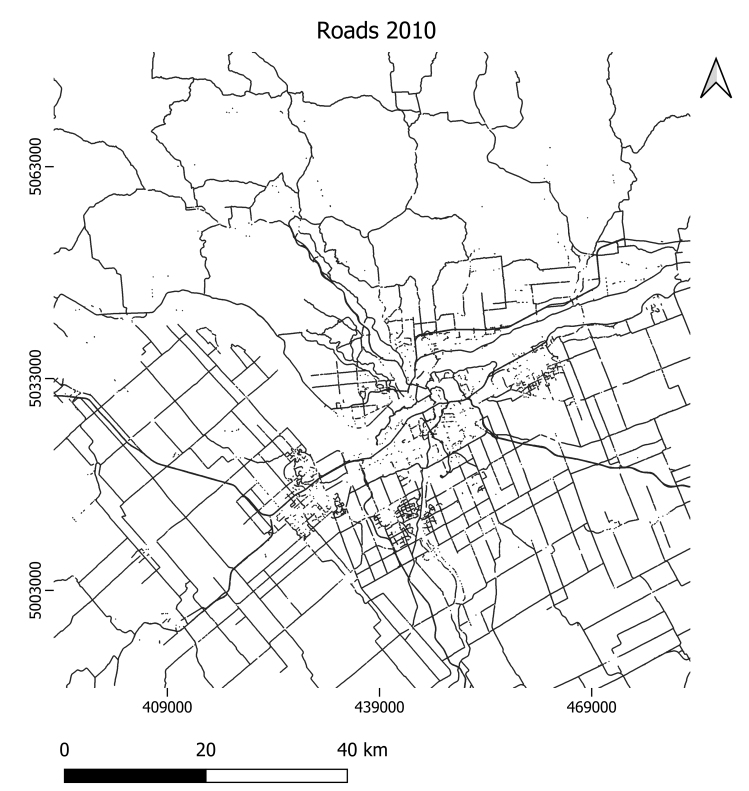

Supplement: S11 Fig — (TIF) [file pone.0328656.s011.tif]

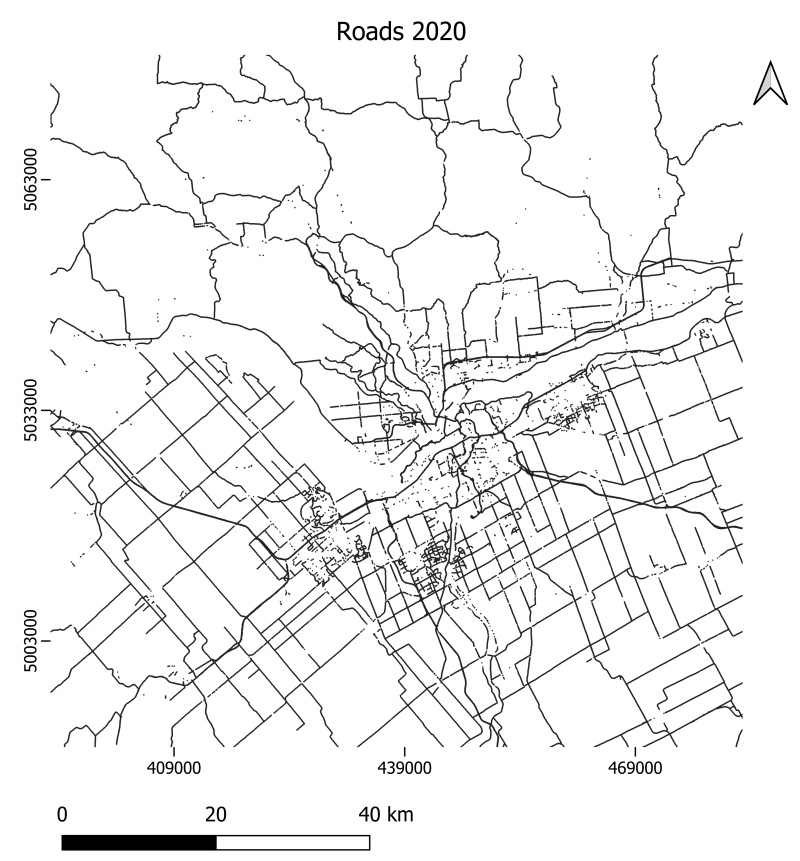

Supplement: S12 Fig — (TIF) [file pone.0328656.s012.tif]
