# Supplementary material for: MSPA-informed SLEUTH urban growth modeling for green space protection in Ottawa
Source: PLoS One. 2025 Aug 8;20(8):e0328656. doi: 10.1371/journal.pone.0328656 (PMC12334021; doi:10.1371/journal.pone.0328656)
Supplement: Attachment — Submitted filename: Responses to the reviewers2.docx [file pone.0328656.s014.docx]

**Answers to the reviewers’ comments**

**Dear Editor,**

As previously stated, all figures (1 to 6) in our paper were obtained using open-access and freely available sources. Figure 1 is a Landsat ETM+ color composite image, which was downloaded from Earth Explorer, a platform providing these images free of charge. Figures 2 to 6 were produced as part of our analysis of the area and are our original work. Where external data sources were used to enhance the presentation, such as the inclusion of roads, we have appropriately referenced sources like OpenStreetMap (OSM), which is publicly accessible and free to use.

For the figures in the appendix, the slope and hillshade layers were generated using freely available CDEM data, for which we have also provided appropriate references in the text. The land use layers were produced using data from GLC_CFS30, Dynamic World, and Gaia, all of which are freely accessible and have been properly cited in the text. The urban extent layers in the appendix were derived from the land use layers, and we have already explained and referenced their sources within the main text. Additionally, the road layers in the appendix were extracted using data from OSM, which is freely available, and we have ensured sufficient reference to these sources in the text.

We hope this explanation is sufficient for you to proceed with sending the paper to reviewers if you deem it appropriate.

Best regards

The authors
