# Supplementary material for: MSPA-informed SLEUTH urban growth modeling for green space protection in Ottawa
Source: PLoS One. 2025 Aug 8;20(8):e0328656. doi: 10.1371/journal.pone.0328656 (PMC12334021; doi:10.1371/journal.pone.0328656)
Supplement: Attachment — Submitted filename: Responses to the reviewers_2.docx [file pone.0328656.s015.docx]

**Answers to the reviewers’ comments, Second Round**

We would like to thank the respected editor and reviewers for their thoughtful comments and suggestions, which have helped improve the quality of our manuscript. We have carefully considered all the editor’s and reviewers' comments, and below are our responses to them:

- We have provided both a highlighted and non-highlighted version of the revised manuscript.
- We have addressed all comments to the best of our ability.

**Reviewer #2:**

**Comment 1**. The innovation and contribution of the paper need to be further highlighted. The paper's innovations need to be further emphasized, such as: Is it the first time that MSPA core importance and connectivity are integrated into the SLEUTH-3r model?

**Response**:

As far as the length of the paper allowed, the innovation and uniqueness of the study are highlighted in lines 46-47, 107-109 and 122-126.

**Comment 2**. The research hypothesis and objectives are not clear enough. It is suggested to supplement the following contents: The main assumptions of the study (e.g., can green space protection be achieved through scenario optimization?). Clear research objectives (such as proposing green space protection strategies applicable to rapidly urbanized areas).

**Response**:

The required sentences as to the objectives and assumptions of the study have been added to the paper in lines 113-119.

**Comment 3**. The data sources and processing are not clear. For example, how to ensure the consistency between different data sources according to GLC_CFS30, Dynamic World and GAIA classification standards and reclassification details?

**Response**:

Regarding consistency between data sources, lines 144–147 of the paper explain that only the urban areas from Dynamic World and GAIA were used to supplement and compare the urban areas in the GLC-CFS30 dataset. Therefore, it was not necessary to align the different classification schemes used by these sources.

**Comment 4**. The construction of the exclusion layer requires more detailed description. Exclusion layers Exclusion 1 and Exclusion 2 are the key to the study, but the paper describes their construction process vaguely. The differences between the two exclusion layers and their specific impact on the results need to be discussed in depth.

**Response**:

In lines 173-177, we have provided more detailed explanation of the differences between the two exclusion layers.

**Comment 5**. Although the calibration of SLEUTH-3r is mentioned to use OSM indicators, the calibration steps and parameter Settings are not explained in detail. It is suggested to supplement the weight and selection basis of each index in the calibration process. It is suggested to discuss the applicability of ROC and PR results, and whether there are other indicators that can be supplemented (such as Kappa coefficient).

**Response**:

As far as the length of the paper allowed, more information on calibration steps has been added in lines 235-242. Although there are other methods to assess the accuracy of the results, we have mentioned in lines 247-253 that in our case, using ROC and PR are more appropriate than Kappa coefficient.

**Comment 6**. Although the paper puts forward 8 scenarios, it does not discuss the limitations and applicability of each scenario. For example, will "compact growth" lead to social and economic imbalances in some regions? How scientific is the distribution basis and adjustment method of social equity weight? It is suggested to supplement the discussion.

**Response**:

In lines 264-275 of the paper, we have added explanation of how we have generated compact growth and its implications. In lines 280-287, and 289-290 we have further explained the process and the need to future studies as to the imbalances that may occur as a result of putting the compact city and social equity scenarios into effect.

**Comment 7**. Some figures lack clear explanations (such as the meaning of colors and symbols in Figure 4-6), which may lead to difficulties in understanding. The numerical comparison in table 3 is clear, but lacks a prominent description of key results.

**Response**:

In lines 351-358, we have added explanation of the meaning of colors and symbols in Figures 4-7 (old numbers: Figures 4-6). Also, in lines 318-323, more description has been given for key results of Table 3.

**Comment 8**. The keywords are too much. I suggest remove the Ontario, Scenario.

**Response**:

Two keywords have been removed.

**Comment 9**. In line 92, the authors mentioned the appropriate resolution of input images for the model to function effectively remains a research gap. Is this gap solved in your research?

**Response**:

In lines 310-311 and 461-464 of the paper, we have explained the appropriate resolution of the input images for our study area. Further in-depth analysis of this aspect requires more technical explorations to be covered in future studies.

**Comment 10**. The sub-title 2.1 should be revised to study area. The 2.2 should be revised to Data source. The 2.3 could revised to Methods.

**Response**:

The required revisions have been implemented.

**Comment 11**. In line 134-135, this area has recently undergone rapid urban growth, threatened green spaces and justified the need to focus on future city growth projections and effective management strategies. It should be supplied with data.

**Response**:

In line 137, we supplied quantitative data as to the rapid urban growth that has occurred in our study area.

**Comment 12**. I have a question, the earliest OSM street is 2014, how to get 1990,2000,2010 road network? It should be illustrated clearly.

**Response**:

Thanks for your comment. In lines 179-186, we have added further explanation as to how the road layers for the earlier years have been generated.

**Comment 13**. I suggest to add a technique map in method section to make it clear. In line 330, a sentence as a paragraph is not suitable.

**Response**:
We have added a flowchart map of the study as Figure 4. We hope this figure provides clear description of the steps taken in our study.

**Comment 14**. The content should correspond to specific figures or tables. Such as line 283-287, and line 305-310.

**Response**:
Thanks for your comment. We have addressed these issues.

**Comment 15**. The language and expression problems. Some sentences are too long and not concise enough, such as the introduction and methods sections. It is recommended to further polish the language to ensure that the expression is concise and logical.

**Response**:

We have tried to address this issue by making some the long sentences shorter and polishing the language. Hope the paper is now more readable.

**Comment 16**. The references cited are not standard enough. For example, the section 2.2 data source, this section is not necessary cited references. The references should not repeat shown many times in this manuscript such as line 212 and 213, the number should be in sequence like 1,2,3....50. The citation in line 156-157,165 also not necessary.

**Response**:

We have removed the unnecessary references and their repetitions in the paper.

**Comment 17.** Some important references should be cited in line 59-60 as follow. Spatiotemporal patterns of vegetation phenology along the urban-rural gradient in Coastal Dalian, China. Urban Forestry &Urban Greening, 2020,54,126784. doi:10.1016/j.ufug.2020.126784, Spatiotemporal variation characteristics of green space ecosystem service value at urban fringes: A case study on Ganjingzi District in Dalian, China. Science of The Total Environment, 2018,639:1453-1461.doi:https://doi.org/10.1016/j.scitotenv.2018.05.253, Spatial and temporal heterogeneity of urban land area and PM2.5 concentration in China. UrbanClimate,2022,45:101268. doi:https://doi.org/10.1016/j.uclim.2022.101268. Spatial influence of exposure to green spaces on the climate comfort of urban habitats in China. UrbanClimate,2023,51:101657. doi:https://doi.org/10.1016/j.uclim.2023.10

**Response**:

Thanks for your comment. We have now included the above references numbered 6-9 in our paper.

**Reviewer #3:**

**Comment 1**. Further emphasize the article's innovation points in the introduction or discussion section and supplement existing related research more extensively. For example, the authors could reference the role of rooftop greening and pocket parks (10.1016/j.scs.2025.106261, https://doi.org/10.1016/j.foar.2023.12.007) as well as the benefits of protecting green corridors for urban ecology (10.3390/land11020165,10.1016/j.apgeog.2024.103439).

**Response**:

As far as the length of the paper allowed, the innovation and uniqueness of the study are highlighted in lines 46-47, 107-109 and 122-126. We have also included the mentioned references in our paper, now numbered 58-61.

**Comment 2**. Is collecting land use data every ten years for prediction too coarse? In the methods section, provide reasons for the selected time points, such as data availability and the validity of the time span for urban growth prediction. Would more detailed temporal classifications improve model accuracy?

**Response**:

As explained in lines 147–149, the 10-year interval for the input images was selected primarily for two reasons: the availability of data and the need for a sufficient period to capture detectable urban growth in the imagery.

**Comment 3**. How would using higher-resolution data (e.g., 30 meters or 10meters) affect model results? Could future research be suggested to verify this?

**Response**:

In lines 310-311 and 461-464 of the paper, we have explained the appropriate resolution of the input images for our study area. Further in-depth analysis of this aspect requires more technical explorations to be covered in future studies. We have mentioned this need in lines 461-465.

**Comment 4**. What is the definition of a compact city scenario? How can it be more accurately quantified? Suggest adding a clear definition of a compact city in Section 2.3.7, for example: "A compact city emphasizes high-density development, mixed land use, and efficient transportation systems."

**Response**:

In lines 264-275, we have explained “Compact Scenario”. Thanks for your comment.

**Comment 5**. Explain the rationale for simulating compact cities by adjusting diffusion, breed, road gravity, and slope resistance coefficients in the SLEUTH-3r model.

**Response**:

In lines 264-275, we have explained the rationale for simulating Compact Scenario by adjusting the SLEUTH-3r coefficients.

**Comment 6**. How are the weights in the TOPSIS method assigned? In Section 2.3.9, describe the weight allocation method in detail and explain the rationale for assigning weights to each indicator, such as why the affected core area is given the highest weight. Compare this with other methods like the Analytic Hierarchy Process (AHP) or entropy weight method.

**Response**:

In lines 377-389 we have added more explanation of the weights used in the TOPSIS and the rationale behind these decisions. Also, more information on the preferences given to the green space cores has been provided in these lines.

**Comment 7**. The authors mention sensitivity analysis of weights (e.g., the 20,000 iterations mentioned in the article); please further explain its impact on the results.

**Response**:

As far as the length of the paper allowed, more information on sensitivity analysis in the TOPSIS has been provided in lines 377-389. Thanks for your comment.

**Comment 8**. What is the rationale for selecting green space cores (including cores, perforations, and edges)? Is there sufficient justification? Suggest supplementing the explanation of the MSPA classification method and its application in selecting green space cores in Section 2.3.1. Cite relevant literature to support the rationale for selecting cores, perforations, and edges, such as their significance to ecosystem connectivity.

**Response**:

In lines 96-98 and 160-161, more explanation has been provided on MSPA and the rationale for selecting green space cores. In these lines, we have also provided due reference justifying our practice.

**Comment 9**. In the conclusion section, expand the discussion on future research directions, such as how to optimize the model by incorporating more socio-economic factors and policy changes. Further clarify the practical application value of the article, such as providing scientific guidance for urban planners and policymakers, for example, what protection strategies should be adopted in different regions and whether these strategies have room for dynamic adjustments.

**Response**:

In lines 464-470, we have provided discussion on future research directions and in lines 471-477, we have indicated the applications of the research. Thanks for your comment.

Once again, we appreciate the reviewers' detailed feedback and believe the revisions have strengthened the manuscript. We hope these changes meet the reviewers' expectations. We believe our manuscript will provide useful information on application of SLEUTH-3r and inclusion of green space information into the process, especially for large areas with dispersed urban growth. We are happy to make further changes if the esteemed reviewers deem them necessary.

Best regards

The authors
