# Supplementary material for: MSPA-informed SLEUTH urban growth modeling for green space protection in Ottawa
Source: PLoS One. 2025 Aug 8;20(8):e0328656. doi: 10.1371/journal.pone.0328656 (PMC12334021; doi:10.1371/journal.pone.0328656)
Supplement: Attachment — Submitted filename: Responses to the reviewers_3.docx [file pone.0328656.s016.docx]

**Answers to the reviewers’ comments, Third Round**

We would like to thank the respected editor and reviewers for their thoughtful comments and suggestions, which have helped improve the quality of our manuscript. We have carefully considered all the editor’s and reviewers' comments, and below are our responses to them:

- We have provided both a highlighted and non-highlighted version of the revised manuscript.
- We have addressed all comments to the best of our ability.

**Reviewer #2:**

**Comment 1**. Although the paper mentions the first combination of MSPA and SLEUTH-3r, it does not fully elaborate how to expand the existing SLEUTH-3r application scope, or how to fill the gap of MSPA in urban expansion modeling?

**Response**:

Within the constraints of the paper’s length, references to expanding the scope of the SLEUTH-3r application were added in lines 87–88, 94–95, and 420–422.

**Comment 2**. The SLEUTH-3r calibration step has been added, there is still a lack of detailed discussion on how to adjust the key parameters (such as Diffusion, Breed, Road Gravity, etc.), especially how to derive these parameters from actual geographical conditions.

**Response**:

Within the allowed length of the paper, we included a description of how to adjust the coefficients of the SLEUTH-3r model in lines 218–225.

**Comment 3**. The paper mentions that "compact cities" may lead to social and economic imbalances, but the specific suggestions are not mentioned. For example, how different income groups will be affected?

**Response**:

The effects of model results on income groups warrant separate future studies and fall beyond the scope of this paper. However, we have acknowledged these limitations in the Methods, Discussion, and Conclusion sections (lines 274, 450–451, and 475–477) to guide interested researchers.

**Comment 4**. It is lack of green space protection policy of different regions (such as urban core area and suburbs), and there is a lack of more targeted practical guidance.

**Response**:

Regarding practical guidance, we believe the maps generated in our study offer a strong foundation for supporting green space protection in Ottawa’s urban planning. However, we acknowledge that detailed, on-the-ground decisions require finer-scale data and consideration of additional factors. Our results currently suggest that urban growth is more suitable along the northeast–southwest axis of the region. We have elaborated on this in lines 45–46, 358–369, and 411–412.

**Comment 5** Some paragraphs in the paper are too long (such as the method section), which affects the fluency of reading. For example, Introduction and Methods sections can be further compressed to highlight the key points.

**Response**:

We have revised and shortened the paragraphs in the Introduction and Methods sections to improve clarity and readability.

**Comment 6**. The symbol interpretation of some charts (such as Figure 5-7) is still brief, and the spatial differences of different scenarios are not intuitively reflected.

**Response**:

We have clarified the interpretation of Figures 5–7 and explained the symbols used in lines 358–369 of the manuscript.

**Reviewer #3:**

No further comments have been suggested by the respected Reviewer #3.

Once again, we sincerely appreciate the reviewers’ detailed feedback, which we believe has significantly improved the manuscript. We hope the revisions meet the reviewers’ expectations. Our study offers valuable insights into the application of SLEUTH-3r and the integration of green space data, particularly for large regions experiencing dispersed urban growth. We respectfully hope the revised manuscript will be found suitable for publication in your esteemed journal.

Best regards

The authors
